# Improving health information systems for decision making across five sub-Saharan African countries: Implementation strategies from the African Health Initiative

**DOI:** 10.1186/1472-6963-13-S2-S9

**Published:** 2013-05-31

**Authors:** Wilbroad Mutale, Namwinga Chintu, Cheryl Amoroso, Koku Awoonor-Williams, James Phillips, Colin Baynes, Cathy Michel, Angela Taylor, Kenneth Sherr

**Affiliations:** 1 Centre for Infectious Disease Research in Zambia, Zambia; 2 School of Medicine, University of Zambia, Zambia; 3 Partners In Health/Inshuti Mu Buzima, Rwanda; 4 Upper East Regional Health Directorate, Ministry of Health, Ghana; 5 Heilbrunn Department of Population and Family Health, Mailman School of Public Health, Columbia University, NY, USA; 6 Ifakara Health Institute , Mikocheni, Dar-es-Salaam, Tanzania; 7 Health Alliance International, Direcçao Provincial de Saúde, Beira, Sofala, Mozambique; 8 Department of Global Health, University of Washington, Seattle, USA

## Abstract

**Background:**

Weak health information systems (HIS) are a critical challenge to reaching the health-related Millennium Development Goals because health systems performance cannot be adequately assessed or monitored where HIS data are incomplete, inaccurate, or untimely. The Population Health Implementation and Training (PHIT) Partnerships were established in five sub-Saharan African countries (Ghana, Mozambique, Rwanda, Tanzania, and Zambia) to catalyze advances in strengthening district health systems. Interventions were tailored to the setting in which activities were planned.

**Comparisons across strategies:**

All five PHIT Partnerships share a common feature in their goal of enhancing HIS and linking data with improved decision-making, specific strategies varied. Mozambique, Ghana, and Tanzania all focus on improving the quality and use of the existing Ministry of Health HIS, while the Zambia and Rwanda partnerships have introduced new information and communication technology systems or tools. All partnerships have adopted a flexible, iterative approach in designing and refining the development of new tools and approaches for HIS enhancement (such as routine data quality audits and automated troubleshooting), as well as improving decision making through timely feedback on health system performance (such as through summary data dashboards or routine data review meetings). The most striking differences between partnership approaches can be found in the level of emphasis of data collection (patient *versus* health facility), and consequently the level of decision making enhancement (community, facility, district, or provincial leadership).

**Discussion:**

Design differences across PHIT Partnerships reflect differing theories of change, particularly regarding what information is needed, who will use the information to affect change, and how this change is expected to manifest. The iterative process of data use to monitor and assess the health system has been heavily communication dependent, with challenges due to poor feedback loops. Implementation to date has highlighted the importance of engaging frontline staff and managers in improving data collection and its use for informing system improvement. Through rigorous process and impact evaluation, the experience of the PHIT teams hope to contribute to the evidence base in the areas of HIS strengthening, linking HIS with decision making, and its impact on measures of health system outputs and impact.

## Background

Health Information Systems (HIS) are one of the six essential and interrelated building blocks of a health system. A well-functioning HIS should produce reliable and timely information on health determinants, health status and health system performance, and be capable of analyzing this information to guide activities across all other health system building blocks [1]. Thus, an HIS enables decision-makers at all levels of the health system to identify progress, problems, and needs; make evidence-based decisions on health policies and programs; and optimally allocate scarce resources [2-4] – all of which are key elements in the success of large-scale efforts to achieve health improvements [5].

Weak HIS are a critical challenge to reaching the health-related Millennium Development Goals [6,7]. Evaluations of routine health facility data have identified consistent problems in HIS completeness, accuracy and timeliness in low- and middle-income country (LMIC) health settings [8,9], which limit HIS use for routine primary health care (PHC) planning, monitoring, and evaluation [10-12]. Other factors associated with poor quality data in resource constrained settings include duplicate, parallel reporting channels and insufficient capacity to analyze and use data for decision making [13].

Improving HIS functioning is a priority given its central role in the delivery of equitable and high quality health services, though approaches to improving HIS vary. Simple data quality assessments that engage frontline health workers and data managers have been used to verify, standardize, and improve routine HIS data [14-16]. Other approaches have focused on technological interventions such as information communication technologies (ICT) designed to reduce errors through reducing data bulkiness and automating data collection, validation, and analysis [4,17,18].

To ensure that HIS contribute to improved health services, it is essential that policy makers and health system managers utilize available information for ongoing monitoring of plans and programs, as well as for resource allocation purposes. Information management is a basis for the production of knowledge and its translation for health system decision making [19-21]. Further evidence is needed on effective strategies for linking data system improvements with decision making, including its impact on the delivery of health services and population health.

The Doris Duke Charitable Foundation launched the African Health Initiative to catalyze significant advances in health systems strengthening through supporting Population Health and Implementation Training (PHIT) Partnerships in five sub-Saharan African countries (Ghana, Mozambique, Rwanda, Tanzania and Zambia) [29]. All five PHIT Partnerships include approaches to strengthen the HIS building block as a means of improving health service delivery and, ultimately, population level health. Despite the common goal of improving data capture to support timely decision making, each partnership uses project-specific strategies to strengthen HIS and improve decision making and to target different levels of the health system, including health managers, clinicians, and the community. The full description of each partnership’s methodology is described elsewhere [30-35].

This paper describes, compares, and contrasts the five PHIT Partnership approaches to strengthen HIS and promote the use of data for decision making, focusing on the designs, activities, and the adaptations during the implementation process.

### PHIT Partnership approaches to improve HIS and decision making

Table 1 summarizes the range of models to improve HIS across the five PHIT countries, focusing on integration approaches with the MOH’s HIS, strategies for improving data quality, procedures for handling and manipulating data, strategies for linking data to decision making, and sustainability plans.

**Table 1:** PHIT Partnership health information system innovations

Health Information System Domain	PHIT Partnership Country				
	
	Ghana	Mozambique	Rwanda	Tanzania	Zambia
**Summary**	Register simplification.	Improving quality of MOH’s routine HIS.	EMR.	Community health information system.	EMR using mobile phone technology.

**Integration with national HIS**	Harmonizes data from routine MOH facility forms.	Focuses on national MOH information system (*Módulo Básico*).	Integrated into health information system, national roll-out ongoing.	Not currently integrated.	Not currently integrated.

**Strategy for data quality improvement**	Simplified data capture and streamlined reporting designed to lead to more time to focus on quality.	Ongoing feedback on missing data and outliers, and ongoing data quality assessments across facility, district and provincial levels.	Quarterly data quality audits and automated data quality report based on logic errors generated when administrative and clinical reports are developed.	Facility supervisors review community health agent reports and provide data feedback.	Standardized protocols for data capture with real-time query of data gaps; subsequent follow-up during monitoring visits.

**Levels at which data are used**	Community, health facility and district levels.	Health facility, district and provincial levels.	Community, health facility, district and national levels.	Community, health facility and district levels.	Community, health facility and district levels.

**Data manipulation**	Data are aggregated at sub-district, district, and regional levels, and reported to the national level.	Facility and district level graphs and tables routinely updated for Primary Health Care services.	Data are aggregated and summarized to provide summary indicators.	Data are summarized in tables and graphic forms to facilitate trend analysis.	Data are aggregated and summarized into reports and graphics for easy interpretation.

**Linkage with decision making**	Data used to identify priority areas, and guide planning and resource allocation.	Trend analysis at facility, district and provincial levels to identify priority problems, monitor implementation of modifications, and link with district activity plans and budgets.	Data used by clinicians to plan patient management, as well as district and health facility managers to identify service quality gaps.	Data used for community problem-solving and planning, and incorporated into facility and district planning.	Focus on data use by Community Health Workers to identify patients for follow-up, as well as clinicians and facility managers for performance assessment and improvement.

**Sustainability plans**	Routine use by MOH managers facilitates ownership and continuity.	Integration with current MOH HIS facilitates adoption and continued use of tools and approach.	The EMR has been incorporated into the national HIS.	Demonstrating feasibility and utility of approach expected to generate support for sustaining the approach.	Training all health workers in the intervention area and close relationship with district managers to build HIS ownership.

### Ghana

**Figure 1:**
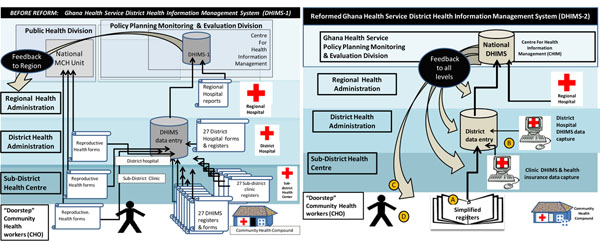
Visual framework for the health information intervention - Ghana

The Ghana PHIT Partnership (the Ghana Essential Health Intervention Project, or GEHIP), has two intervention strategies to strengthen the HIS and link information with improved health system operations. The first is to implement a simplified information capturing system as part of the District Health Information Management System (DHIMS-2) that focuses on essential information for district level planning, thereby reducing the reporting burden in primary care settings (Figure 1). The second is the adoption of a District Health Planning and Reporting Toolkit (DiHPART) for use by district health leadership to identify and allocate resources based on the district level burden of disease profile.

### Rationale and contextual appropriateness

#### Data capture for DHIMS-2

Simplified registers were introduced to standardize data sources, and to ensure consistent supply of registers for community health officers (CHOs). The simplified registers also allow health facilities to rapidly tally figures for monthly summary reports in order to address complex data capture responsibilities that occupied more frontline staff time than clinical service delivery[22]. Prior to the adoption of the simplified registers, maintaining patient encounter registers was complex and cumbersome, involving 27 register books to collect information on patient attendance at outpatient consults, maternity, well-child care, family planning, and home visits. Collating and reporting health information was particularly tedious for CHOs, who record, compile, and report client encounters to sub-district and district levels.

#### Planning and budgeting with DiHPART

Based on the observation that national decentralization policies lack appropriate training and tools for district leaders to base priorities on need, the DiHPART tool was developed to assist managers with planning. As a means of basing decision-making on known patterns of risk, DiHPART removes the guesswork from budgeting, simplifying the task of strategic leadership for health resource allocation.

### Activities and feedback mechanism

#### Data capture for DHIMS-2

The GEHIP team worked with district and sub-district managers and CHOs to review, redesign, and implement the improved versions of the simplified registers over a one-year period. A detailed review was carried out to inventory baseline data collection (data fields collected, registers used), identify redundant information, and assess data collection for appropriateness and relevance for district health managers and CHOs. The physical size of the simplified registers was reduced to make them easier to carry during outreach activities. In the course of this iterative process, the simplified registers were piloted in one district and subsequently adapted to the need of all three GEHIP districts after feedback from CHOs and district health information officers. The data fields collected are regularly reviewed to keep them up to date with those collected by the Ghana Health Service. Procurement, distribution, and content revision functions have been fully integrated into the Upper East Regional Health Information Unit, which facilitates rapid adaptation, adoption, and continued use.

In their final format, the simplified registers include five registers for CHOs to gather data on facility consults for outpatient, maternal and child care services, and outreach services in homes and schools. Although the initial goal was to develop a single register, delineation of functions within health facilities required five registers to collect clinical data when staff were deployed to outreach activities. To ensure data quality and its use, monthly and quarterly data validation meetings are held by CHOs, sub-district, and district teams to review data collected and identify gaps. Subsequently, the data are compiled and submitted to the regional and national levels.

#### Planning and budgeting with DiHPART

DiHPART’s introduction included an orientation for district health management teams to provide an overview of the disease burden and its implications for current plans and activities, followed by identification of adaptations to align spending priorities with risk patterns. Disease burden models for DiHPART were based on cause of death data from locally derived data provided by the Navrongo Health Research Centre.

### Adaptation and learning during implementation

#### Data capture for DHIMS-2

Qualitative appraisal of reactions to the simplified register system suggests that CHOs welcome the reduced documentation burden and additional time for service and outreach. Essential for the register simplification process has been coordination with national HIS reform (Figure 1), including streamlining data collection and aggregation operations (pathway A) , simplifying and computerizing feedback to all levels (pathway C), and enabling health workers to view data feedback and compare performance with counterparts (pathway D).

GEHIP experience has identified additional areas for improvement. Efforts to use cell phone technology for data entry encountered technical problems. In addition, district and regional funds are insufficient to independently cover the recurrent supply cost, including CHO registers. This problem may be resolved when the simplified registers are adopted for nationwide implementation.

#### Planning and budgeting with DiHPART

The experience with implementing DiHPART has differed from expectations in multiple ways. The lack of flexible funds due to earmarked wages and donor requirements has led to a disconnect between DiHPART plans and actual expenditure, which has impeded implementation of DiHPART guided decision making. However, during its implementation, DiHPART has become an influential resource mobilization tool, providing district managers with evidence to lobby political officials for additional resources.

## Mozambique

**Figure 2:**
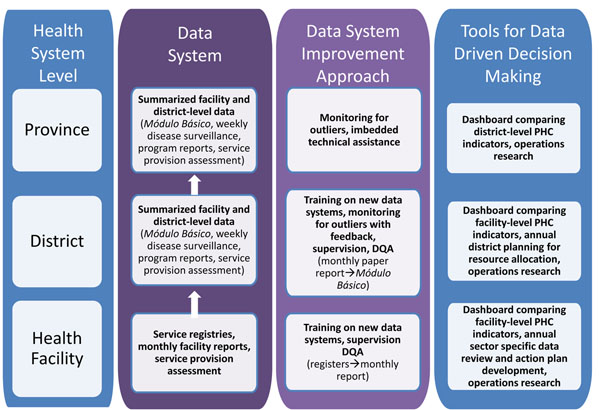
Visual framework for the health information intervention - Mozambique

The Mozambique PHIT strategy focuses on strengthening the MOH’s established HIS through applying innovative approaches to improve HIS quality and foment its use for resource allocation, program monitoring, and service delivery improvements at the facility, district, and provincial levels (Figure 2). The Mozambique project has introduced simplified tools based on routine HIS data to highlight service delivery performance success and problems at the facility and district levels. The project team mentors district and facility health managers to use these tools for identifying, implementing and evaluating efforts to improve health system performance.

### Rationale and contextual appropriateness

The PHIT strategy is designed to work within the MOH priorities, specifically to strengthen the quality and use of the existing information system (*Módulo Básico*). The partnership has adopted and modified nationally developed training modules and data assessment approaches in developing an intervention that is contextually appropriate for district managers.

The PHIT strategy endeavors to improve HIS quality from the facility, district, and provincial levels in Sofala province. Strengthening data for decision making focuses on the district level – the key management unit to support and monitor service delivery improvements at the facility level. Under the government of Mozambique’s decentralization program, district managers are increasingly responsible for resource allocation (including financial and non-financial resources, such as human resources), as well as monitoring and evaluating program activities. The PHIT strategy therefore builds district capacity for using data for decision making and supports their linkages with health facilities to lead to health system improvements.

### Activities and feedback mechanism

Data quality includes training and supporting district and provincial statistics personnel to continuously monitor the performance of the HIS and the provision of timely feedback to facility and district managers to lead to incremental improvements in HIS quality. Furthermore, an annual data quality assessment (DQA) for primary health care (PHC) services is carried out in all districts in the PHIT intervention province, with feedback provided to district and health facility managers via a summary data quality ranking tool that acknowledges facilities with high data quality and identifies facilities with poor data quality for follow-up by health system managers and PHIT-supported personnel [32]. After health facilities with glaring or persistent data quality problems are identified (those in the lowest category of the ranking process), district and provincial health managers provide supportive supervision to facility managers and staff that includes a re-introduction to the HIS and associated tools, clarification of timing and procedures for reporting, and reinforcement of the importance of the HIS. Technical and financial support is also provided to develop and maintain infrastructural capacity to computerize facility summary reports at the district level and send them electronically for monthly collation at the provincial level.

Identifying problems and making informed decisions based on up-to-date data from the HIS is promoted at the facility, district, and provincial levels. District and facility managers are trained and mentored to build competencies and routine practices for basic data analysis, including indicator development and secular trend analysis. Simple tools and graphical representations using routinely collected data have been developed, field tested, and implemented for health system managers to use for monitoring primary health care indicators, target interventions, target resources at the district (to improve facility performance), and provincial levels (to improve district performance) [32] and evaluate whether interventions have led to intended service delivery improvements.

### Adaptation and learning during implementation

During the six-month planning grant, the Mozambique PHIT Partnership piloted and refined a province-specific DQA methodology, which are now in use [14]. Annual assessment results are disseminated to health facility, district, and provincial managers using a simplified ranking system that was developed based on suggestions from a provincial data quality feedback session. Tools to summarize and regularly compare key PHC indicators across facilities and districts have evolved in design and content over the first three years of implementation to include fewer indicators and focus on secular trend analysis and graphic comparisons among peer facilities and districts. Efforts to promote use of data for decision making have also evolved to go beyond training health managers in data systems, indicator development, and analysis approaches. Periodic district-level review and planning meetings bring together peer facility staff with district and provincial leadership to promote active data review combined with planning and monitoring of plan implementation with key stakeholders.

### Rwanda

**Figure 3:**
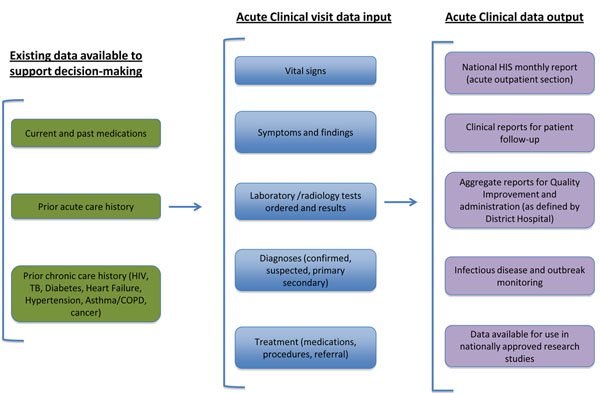
Visual framework for the health information intervention - Rwanda

In Rwanda, the Ministry of Health (MOH) and Partners In Health (PIH) have co-developed an electronic medical record (EMR) system (OpenMRS)[23] and are implementing an enhanced version as part of the PHIT Partnership (Figure 3). In the three PIH-supported districts of Rwanda the EMR holds patient records for 33 health centers, including a catchment area of approximately 800,000 people. The EMR system includes comprehensive medical records for all patients with HIV, tuberculosis, heart failure, epilepsy, hypertension, asthma, chronic obstructive pulmonary disease, diabetes, and cancer. In addition, a medical record system has been developed and is being implemented for acute outpatient consults, including registration, presentation, diagnosis, laboratory tests, and treatment. The EMR supports patient care by providing clinicians with summaries of patient visits and laboratory test results; through reports of at-risk patients (including those with missed visits, low CD4 counts, unsuppressed viral load, and high HBA1c) [24] and through administrative reports to support clinic management, resource allocation, and quality improvement (QI).

### Rationale and contextual appropriateness

Though hospitals have paper patient charts recording prior admissions and emergency room visits, the primary care facilities in the project area do not have a standardized comprehensive outpatient paper-based record. As a result, acute and chronic medical history is not always immediately available to clinicians during patient consultation, and information does not always flow optimally between the levels of care. The EMR system allows for synthesis and access to patient history from chronic and acute outpatient encounters at both levels of care. In addition to the nationally required HIS reports, key EMR outputs include customized reports for QI, administration, and infectious disease monitoring. At present, patient registration data have been used to identify geographic areas with low access to acute outpatient services, while chronic care reports guide care for patients with chronic conditions (including HIV, TB, diabetes, hypertension, heart disease, asthma/COPD and cancer).

The MOH has commenced implementation of a nationwide comprehensive electronic medical record system, based partly on the partnership’s work. Core work for this included agreement on standard terminology for national use, including symptoms and diagnoses linked to international standards and development of a tested and refined user interface. This collaboration ensures that parallel systems are not created, with one national information system that integrates across EMR components and feeds into national HIS reporting requirements.

### Activities and feedback mechanism

Tools that are being introduced include an electronic patient registration system and an acute patient visit record. Each of these have reports as part of the feedback loop that aggregate data at the facility and district levels (for reporting and administrative purposes), as well as the individual patient level for QI and patient tracking purposes. Training is conducted for data officers and coordinators on a quarterly basis, just prior to the quarterly software releases that deliver new content. Clinicians receive both formal and on-the-job training on using the systems and have a point person from the EMR team to support them.

### Adaptation and learning during implementation

In order to allow for integration with the national implementation, the health information model was revised after the terminology standards were discussed with the national e-Health Technical Working Group. Additionally, a training schedule based around software releases and accompanied by more formalized training materials has been developed based on identified field needs.

### Tanzania

**Figure 4:**
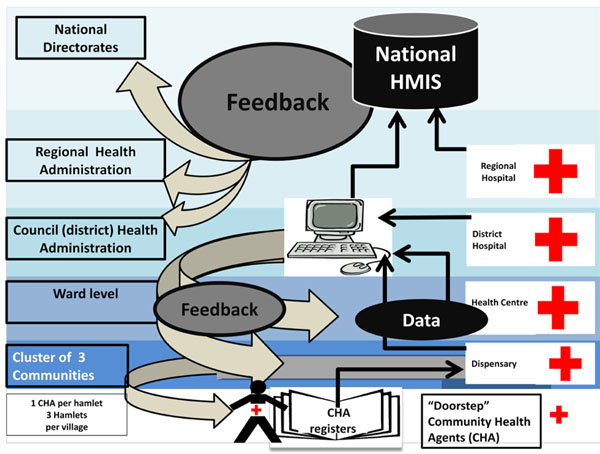
Visual framework for the health information intervention - Tanzania

The Connect Project aims to improve community-level availability, accessibility, and quality of primary health care services using community health agents (CHA) in three districts in rural Tanzania [34]. The Connect Project has adapted and adopted existing community-level health information data capture tools and is working with CHAs to collect and integrate community-level data with the routine HIS at facility and district levels (Figure 4), with data feedback targeting workers at the community, dispensary, health center, and hospital levels.

### Rationale and contextual appropriateness

Although the MOH has developed community-level data collection tools, integrating collected data into the MOH HIS (MTUHA) has been challenging. Facility-based health workers are intended to use the community-level module (MTUHA III) to collect information on a range of community health indicators and report to their corresponding council health management teams (CHMT), who use this information to design an accurate profile of their district and develop Comprehensive Council Health Management Plans. Currently, MTUHA III is not fully or uniformly operative throughout the country owing to a range of systems factors, including workforce shortages that prevent timely and frequent community outreach. The CHA represents an opportunity to pilot and refine approaches to integrate community health information to the MTUHA system.

The Connect project supports integration of community data in the national MTUHA in order to improve the comprehensiveness and quality of health information in general and prompt data interpretation, discussion, and problem solving in community settings. Integration efforts have focused on working with CHA clinical supervisors, village leaders, and CHMT MTUHA coordinators to facilitate their administrative ownership over reporting and utilization of service delivery information from CHAs. As health system and community stakeholder support is built, the Connect HIS system will be customized to reflect the data and reporting requirements of the MTUHA HIS.

### Activities and feedback mechanism

Connect staff worked with MTUHA supervisors to develop two community registers (one for service delivery outputs, a second for community mobilization and health education activities) that provide simple project indicators aligned with the MTUHA III modules. Additional health information summary forms were developed for CHAs to record aggregate data from their registers and report each month to supervisors from their community, the health system, and the Connect team.

CHAs and supervisors from both health facilities and village governments meet regularly to review monthly outputs, identify and troubleshoot problems, and plan jointly with the health system. Connect project coordinators, district MTUHA coordinators, and CHA supervisors hold similar meetings quarterly and transfer CHA health information to district and project managers for planning and program improvement.

### Adaptation and learning during implementation

Data feedback to the CHAs was initially delayed due to the evolving nature of the intervention, the large number and geographic dispersion of study clusters, and variation in CHA supervisor leadership qualities and motivation. To overcome these barriers, the Connect team works with CHA supervisors to motivate their involvement and cover transportation costs incurred while making supportive supervision visits to CHA.

There are notable challenges in collecting and using community-based health information. Supervision visits to all CHAs following initial deployment revealed minor problems concerning the uniformity and proper use of the registers. Project staff and supervisors compiled findings from these visits and convened CHAs in the respective study areas in a joint review of the registers to clarify register use. Management of community-based health information has also been a challenge. Though registers are appropriate for recording service delivery information and aggregating data, they did not facilitate CHAs data use for improving client-focused care as they did not capture household and client information, nor qualitative aspects of service encounters that would be useful for follow-up service encounters. Therefore, the project introduced booklets that remain in each village household where CHAs can log more detailed notes from each visit, which has come at a high financial and logistical cost. Patient referrals from CHAs has also been a challenge, as post-referral feedback from health facilities to guide CHA follow-up services has been erratic. To facilitate the CHA/health facility communication, CHAs, supervisors, and referral providers have been provided closed-user phone groups to communicate without incurring costs.

### Zambia

**Figure 5:**
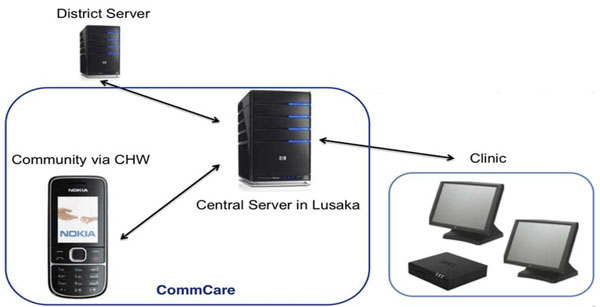
Visual framework for the health information intervention - Zambia

The Better Health through Mentorship and Assessment (BHOMA) project is using an Electronic Data Capture System (EDCS) and mobile technology to improve the quality of data captured in the target districts. The BHOMA system includes a dedicated low-wattage Linux client terminal (powered by solar panels and a 12-volt battery pack) with touch screen data entry terminals attached to a miniature data processing server, into which patient visit information is entered (Figure 5). The system automatically generates performance reports based on predetermined performance indicators that identify facility-level performance gaps and are used by clinical QI teams to mentor facility staff on improving clinical care quality. The EDCS system also automatically generates and sends follow-up messages via general packet radio service (GPRS) technology to CHWs (via mobile phones) to indicate a need for patient follow-up. Using modems and cellular networks, BHOMA clinics access the internet to securely synchronize records to a central server, housed at CIDRZ headquarters in Lusaka, which, in turn, transmits the data to BHOMA district offices, and the MOH’s District Health Offices.

### Rationale and contextual appropriateness

Poor quality data has been a source of concern throughout Zambia and data are frequently not used for evidence-based planning. Furthermore, community-level data are often not collected or used. The expansion of HIV care and treatment in Zambia brought EMR systems to some rural health facilities, which demonstrated their feasibility for capturing patient-level data in real time and their utility in guiding decision making by health system managers. Increases in mobile technology coverage in Zambia has made internet widely available, providing an opportunity to leverage ICT for collection of patient and community level data in real time and to use these data for evidence-based decision making.

### Activities and feedback mechanism

There are six data entry screens (patient registration, adult, pediatric, sick antenatal care (ANC), normal ANC, and labor and delivery) that follow the flow of information on clinical forms. Data are entered and locally and available in real time. To date, BHOMA has trained 72 clinic supporters to enter data for each patient visit and run reports. The five reports include 1) Clinic report (summarizing the number of patient visits at each facility, including follow-up visits for patients with danger signs or severe symptoms who missed their appointment); 2) Patient review report (listing patient charts for the QI teams to review with clinic staff); 3) Clinic performance reports (summarizing twelve clinical care measures for QI teams and clinic staff to use as a snapshot of clinical care quality); 4) CHW performance report (summarizing follow-up and assessment activity levels for CHWs at the health facility); and 5) HIS reports (to remove duplicate burden of tallying data).

Each clinic has a GPRS modem that uses Zambia’s cell phone networks to synchronize de-identified patient records to a central district database every 15 minutes when the system is on. Each district office has a server that aggregates information from all clinics in that district, allowing the QI teams to print patient review and clinic performance reports in preparation for each supportive mentoring visit.

### Adaption and learning during implementation

The BHOMA HIS model has been deployed in largely rural, remote, and understaffed facilities and lessons have become clear during implementation. First, reviewing and clarifying data entry fields reduced the data entry workload. Second, computers with low-power requirements that run on solar power with battery back-up systems are important due to the unreliability of power. Third, using a dedicated client that runs only the BHOMA software avoids viruses, facilitates updates, and simplifies replacement. Fourth, it is essential that clinic performance reports are immediately available at the clinic level — rather than cycling first through the district — for health facility managers to identify areas requiring improvements and to check whether the corrective measures are working. Finally, patient-level information (rather than aggregate data) is used for flagging specific patient charts for follow-up with targeted intervention.

### Comparisons across the PHIT strategies

Although the five PHIT Partnerships have designed different approaches to strengthen health systems in their respective countries, they share common features in enhancing HIS and linking data with improved decision making. Recognizing the complexity and context-specific nature of the intervention settings, PHIT Partnerships have adopted a flexible, iterative approach in designing and refining the development of new tools for HIS enhancement and improved decision making. Across the partnerships, the tools and approaches are designed to actively provide health system performance summaries to enable health system personnel to make informed decision on where to focus their efforts and limited resources. A second common feature is the use of feedback systems to improve data quality, though the error detection and correction approach varies across PHIT Partnerships. Error-detection approaches include automated troubleshooting mechanisms, routine review of aggregate reports for outliers and missing data, or periodic DQAs. A final similarity across PHIT Partnership approaches is the recognition of the importance of MOH information systems to ensure that HIS strengthening efforts are aligned with national priorities and to increase the likelihood of sustained project approaches beyond the life of the African Health Initiative. However, approaches across Partnerships vary in terms of pace and degree of alignment, which can be best described as either front-end integration (Mozambique), progressive integration (Rwanda), current harmonization (Ghana), and potential future harmonization or integration (Tanzania and Zambia).

Despite these similarities, there are notable differences in the PHIT Partnership approaches to HIS strengthening and improved decision making. One difference is the level of focus for data collection, and by extension, its use. The Rwanda, Tanzania and Zambia PHIT Partnerships begin with intensive collection of patient-level data, while the Ghana and Mozambique Partnerships focus on facility, district and provincial-level aggregate data. In addition, the Ghana, Tanzania and Zambia data systems incorporate data from community service provision to direct outreach services from either formal or community health cadres. All systems, however, have sufficient flexibility to manipulate data according to frequency of aggregation (daily, monthly, quarterly, annual), and level of aggregation (health facility, district or province). A second difference is the type of data collection system, with the Rwanda and Zambia Partnerships implementing new EMR systems, while the Ghana, Mozambique, and Tanzania partnerships focus on paper-based HIS that are computerized at the health facility or district levels.

## Discussion

Through the African Health Initiative, the five PHIT Partnerships have designed and are testing novel approaches to enhancing data systems and using HIS results as a driver for decision making and health system performance improvements. Design differences described across the PHIT Partnerships reflect the different theories of change for each project, particularly with regards to what information is needed, who will use the information to affect change, and how this change is expected to manifest. Ghana and Tanzania have simplified paper registries that incorporate data on community service provision, and in Ghana a resource allocation tool pioneered in Tanzania intends to support district managers in decision making. Mozambique focuses on strengthening the existing national HIS, and provides data summaries for health system managers to identify problems, evaluate solutions, and allocate resources. Zambia and Rwanda are implementing ICT approaches to improve data quality, and provide timely information to guide decision making for clinicians and managers. Though implementation of the PHIT interventions is ongoing, there has been significant country-level enthusiasm for building on the HIS innovations of the African Health Initiative, with elements of the programs being adopted nationally in PHIT Partnership countries.

The first three years of PHIT implementation has highlighted a number of elements important for strengthening HIS and linked decision making. First, though an important starting point, training alone is insufficient to engage and build capacity for facility and community health workers. Stakeholder meetings, data reviews, and mentored use of data as a basis for decisions have been utilized to engage health workers and managers and demonstrate the value of data, HIS quality, and ownership of tools to summarize data and guide decision making. A second lesson learned is that it is critical for HIS interventions to be developed in the context of the national HIS, which has been feasible across PHIT Partnerships and is crucial to ensuring sustainability of the programs beyond the project lifespan. Finally, in two of the PHIT Partnerships, the increased availability of mobile phone technology has facilitated the introduction of EMR systems in rural, resource constrained environments. These ICT innovations have come at a high initial financial cost to build infrastructure, modify software, and build human resource capacity for their use.

Like many complex health system interventions, success of the PHIT HIS and decision-making approaches will hinge on whether frontline health workers and managers value, adopt and own the tools and procedures introduced by the country Partnerships [19,21]. For HIS to have an impact on health system functioning, and ultimately population health, it will be the institutionalization of habits and norms around data that will make the difference, such that prioritizing and using quality data is as much a part of routine practice as stocking a pharmacy or immunizing a child. Though exploring different approaches, all PHIT Partnerships are working towards the goal of standardized and routinely used procedures to improve data quality, its availability, and use.

The PHIT Partnerships have both a common evaluation framework and project specific evaluation plan in place to assess their impact on health system functioning and population health [36]. Identifying effective and appropriate strategies for improving data availability, quality and its use, as well as the role of HIS in improving the health service delivery (including the quality and coverage of these services), will contribute to the limited evidence on this health system building block. Taking lessons learned to scale, however, will require substantial investment in general PHC information systems rather than disease specific information systems that can fragment, distort, and weaken country HIS at all levels of the health system [^25^].Without a well-functioning HIS, it is unlikely that the remaining five building blocks of a health system can reach their full potential in improving population health [26-28].

## List of abbreviations used

BHOMA: Better Health through Mentorship and Assessment; CHA: Community health agent; CHMT: Community Health Management Team; CHO: Community health officer; CHW: Community health worker; CIDRZ: Center for Infectious Disease Research in Zambia; DHIMS-2: District Health Information Management System; DiHPART: District Health Planning and Reporting Toolkit; DQA: Data quality assurance; EDCS: Electronic data capture system; EMR: Electronic medical record; GEHIP: Ghana Essential Health Intervention Project; GPRS: General packet radio service; HIS: Health information system; HIV: Human immunodeficiency virus; ICT: Information communication technologies; LMIC: Low and middle income country; MOH: Ministry of Health; MTUHA: MOH health information system in Tanzania; MTUHA III: MOH HIS community-level module in Tanzania; PHC: Primary health care; PHIT: Population Health and Implementation Training; PIH: Partners In Health; QI: Quality Improvement; TB: Tuberculosis.

## Competing interests

The authors declare that they have no competing interests.

## Declarations

This article has been published as part of *BMC Health Services Research* Volume 13 Supplement 2, 2013: Improving primary health care to achieve population impact: the African Health Initiative. The full contents of the supplement are available online at http://www.biomedcentral.com/bmchealthservres/supplements/13/S2. Publication of this supplement was supported by the African Health Initiative of the Doris Duke Charitable Foundation.
